# KGDiff: towards explainable target-aware molecule generation with knowledge guidance

**DOI:** 10.1093/bib/bbad435

**Published:** 2023-12-01

**Authors:** Hao Qian, Wenjing Huang, Shikui Tu, Lei Xu

**Affiliations:** Department of Computer Science and Engineering; Centre for Cognitive Machines and Computational Health (CMaCH), Shanghai Jiao Tong University, Shanghai 200240, China; Department of Computer Science and Engineering; Centre for Cognitive Machines and Computational Health (CMaCH), Shanghai Jiao Tong University, Shanghai 200240, China; Department of Computer Science and Engineering; Centre for Cognitive Machines and Computational Health (CMaCH), Shanghai Jiao Tong University, Shanghai 200240, China; Department of Computer Science and Engineering; Centre for Cognitive Machines and Computational Health (CMaCH), Shanghai Jiao Tong University, Shanghai 200240, China

**Keywords:** molecule generation, diffusion models

## Abstract

Designing 3D molecules with high binding affinity for specific protein targets is crucial in drug design. One challenge is that the atomic interaction between molecules and proteins in 3D space has to be taken into account. However, the existing target-aware methods solely model the joint distribution between the molecules and proteins, disregarding the binding affinities between them, which leads to limited performance. In this paper, we propose an explainable diffusion model to generate molecules that can be bound to a given protein target with high affinity. Our method explicitly incorporates the chemical knowledge of protein–ligand binding affinity into the diffusion model, and uses the knowledge to guide the denoising process towards the direction of high binding affinity. Specifically, an SE(3)-invariant expert network is developed to fit the Vina scoring functions and jointly trained with the denoising network, while the domain knowledge is distilled and conveyed from Vina functions to the expert network. An effective guidance is proposed on both continuous atom coordinates and discrete atom types by taking advantages of the gradient of the expert network. Experiments on the benchmark CrossDocked2020 demonstrate the superiority of our method. Additionally, an atom-level explanation of the generated molecules is provided, and the connections with the domain knowledge are established.

## INTRODUCTION

Structure-based drug design (SBDD) is a highly effective approach in discovering new drug candidates for important protein targets [1] and has gained increasing attention from the academic community. However, it remains challenging to seek appropriate drug candidates for a specific protein target due to the enormous space of valid chemical molecules [2]. Traditional drug design methods, such as virtual screening, molecular dynamics simulations and free energy calculations, are computationally intensive [3] and often lack novelty. Nowadays, researchers have started utilizing deep generative models to design drugs from scratch, which is quite efficient and possesses a great capacity for de novo drug development.

Many target-aware generative models have been developed based on 1D molecular strings [4, 5] or 2D molecular graphs [6]. These methods are effective but limited because it is difficult to capture the patterns of the interaction between proteins and molecules solely from the sequence or 2D graph input. The protein–ligand interaction highly depends on the 3D structures of the protein and the molecule. Nowadays, the advent of dedicated 3D molecular protein binding databases tailored for deep learning, such as CrossDocked2020 [7], has provided new opportunities for a new wave of deep generative models [7–14]. Unlike the aforementioned methods, these approaches explicitly focus on learning the interactions between protein pockets and molecules in 3D space, which is more effective in target-aware drug design. For example, LiGAN [7] voxelizes the 3D space of protein–ligand complexes and employs a 3D CNN to extract atom features within a conditional VAE framework. GraphBP [10], AR [8], Pocket2Mol [9] and FLAG [14] generate atoms or motifs in the 3D space of protein pockets using autoregressive approaches with graph neural networks (GNNs). TargetDiff [13], DiffBP [12] and DiffSBDD [11] utilize diffusion models to generate molecules by iteratively denoising them from noise.

While these methods demonstrate proficiency in generating valid and innovative molecules for binding the specific protein pockets, they still have two limitations. First, the structural information of the target proteins is incorporated into the models by learning the joint distribution of molecules and proteins, and this incorporation is inadequate in guiding the models to generate molecules with high binding affinities, because it is difficult to learn the joint distribution to effectively distinguish between favorable and unfavorable binding gestures of molecule-protein complexes. If the molecules are used as drug candidates, it is crucial for molecules to efficiently and firmly dock with their protein targets [15]. Second, the driving force of the molecule generation to satisfy the desired binding property is usually implicitly produced by the existing model, which is not explainable by chemical domain knowledge.

To overcome the above limitations, we propose a knowledge-guided diffusion model (KGDiff) to generate molecules that are able to bind the given target protein with high affinity. The domain knowledge of protein–ligand binding is distilled and conveyed from the AutoDock Vina [16] scoring functions to our model by fitting them with an SE(3)-invariant expert neural network. The expert network and the denoising network in the denoising process are built to share the representation learning on the atom types and coordinates of the molecule, so that the atom representation learning is enhanced by jointly training the two networks. Moreover, we devise an effective guidance on both discrete atom types and continuous atom coordinates, and then the molecules that binds to the protein target with high affinity are gradually generated in a controlled way during the denoising process. Experimental results on CrossDocked2020 [7] demonstrate the superiority of our approach over the existing state-of-the-art (SOTA) baselines. Furthermore, we provide an atom-level explanation of the generated molecules and establish connections with domain knowledge. Besides, we demonstrate that KGDiff prefers to large pockets with concave surfaces and has the ability to generate molecules with protein subtype selectivity.

## METHODS

### Task definition

Firstly, we designate an atom in the 3D space as $\mathcal{A} = (\boldsymbol{x}, \boldsymbol{z})$, where $\boldsymbol{x} \in \mathbb{R}^{3}$ represents the Cartesian coordinates, and $\boldsymbol{z} \in \mathbb{R}^{K}$ represents the atom features of $K$ dimension. Subsequently, a protein pocket can be defined as $\mathcal{P} = \{ \mathcal A_{P}^{i} \}^{N_{P}}_{i=1}$, and a molecule can be represented as $\mathcal{M} = \{ \mathcal A_{M}^{i} \}^{N_{M}}_{i=1}$, where $N_{P}$ and $N_{M}$ represent the number of atoms in a protein pocket and a molecule, respectively. Given a specific protein $\mathcal{P}$, the objective is to find $\mathcal{M}^{*} = \mathop{\arg \max }\limits _{\mathcal{M} \in \mathbb{M}}f(\mathcal{M}, \mathcal{P})$, where $\mathbb{M}$ denotes a set of all valid molecules in chemical space, and $f$ is a scoring function that indicates the binding affinity of a molecule to a protein.

### An overview of the proposed method

**Figure 1:**
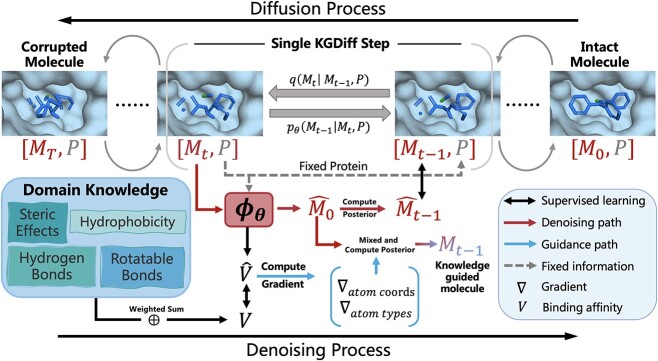
An overview of KGDiff. During the diffusion process, molecules are gradually added predefined noise across ${T}$ time steps. The model $\phi _{\theta }$ is trained to reconstruct molecules from noisy ones and predicts the binding affinities $v$ between the intact molecules $M_{0}$ and the fixed proteins $P$ across a reversed ${T}$ steps. When generating molecules, we further utilize the predicted $\hat{v}$ to guide the denoising process towards high binding affinities. We keep protein structures fixed throughout the whole process.

An overview of the proposed method, namely KGDiff, is given in Figure 1. KGDiff is a diffusion-based generative model. Preliminaries about diffusion models can be found in Supplementary Materials. In the training phase, it takes both a protein and a molecule as input, and uses a forward molecular diffusion process to transform the molecule into a noisy, corrupted one, while keeping the protein target fixed. Then, a denoising process is implemented in the reverse direction to reconstruct the input molecule. The key contribution of KGDiff is to incorporate domain knowledge, i.e. the binding affinity into the denoising process, and guide the process to generate a molecule that has high binding affinity with the protein target.

### Incorporating binding affinity into KGDiff

One way is to compute the binding affinity score externally via AutoDock Vina [16], and apply the score directly into the model. This way has several drawbacks. The score may be used to rank the generated molecules constructed by a post processing step, but it is not able or difficult to affect the denoising process which generates the molecule in a progressive manner. Moreover, it replies heavily on the external docking program which becomes computationally expensive for frequent calls. Last but not least, the docking programs such as Vina usually require the input molecule being valid, which is a difficult condition for the intermediate generation of the diffusion model to satisfy.

In this paper, we propose to distill the domain knowledge in the affinity computation by learning to fit the affinity terms of the Vina functions in Eq. (S1)-(S3) in Supplementary Materials. As given in Figure 1, we devise a two-branch neural network $\phi _{\theta }$ with a shared feature extraction module for both denoising prediction and affinity fitting as follows: 


(1) \begin{align*} [\widehat{\mathcal{M}}_{0}, \hat{v}] = \phi_{\theta}(\mathcal{M}_{t}, t, \mathcal{P}), \: \widehat{\mathcal{M}}_{0} = [\boldsymbol{\hat{x}_{0}}, \boldsymbol{\hat{z}_{0}}], \: \mathcal{M}_{t} = [\boldsymbol{x_{t}}, \boldsymbol{z_{t}}],\end{align*}

where $\widehat{\mathcal{M}}_{0} = [\boldsymbol{\hat{x}_{0}}, \boldsymbol{\hat{z}_{0}}]$ is further used to obtain $\mathcal{M}_{t-1} = [\boldsymbol{x_{t-1}}, \boldsymbol{z_{t-1}}]$ for the next denoising step. $t$ denotes the diffusion step. $\hat{v}$ is the predicted binding affinity for the knowledge-based computation of a weighted sum of the Vina affinity terms with a penalty on the number of rotatable bonds, which computes the binding affinity $v$ (as the ground-truth) between the intact molecule $\mathcal{M}_{0}$ and its protein target $\mathcal{P}$.

Specifically, the neural network $\phi _{\theta }$ is computed first by an SE(3)-equivariant GNN $\phi _{\omega }$ for the representation learning of the atoms, and then by two multilayer perceptron (MLP) functions separately for the atom type and the binding affinity prediction. That is, we have 


(2) \begin{align*} \boldsymbol{\hat{z}_{0}} = \text{softmax}(&\text{MLP}_{1}(\boldsymbol{\hat{h}_{0}})), {\hat{v} = \frac{1}{N_{M}}\sum_{i=0}^{N_{M}-1} \sigma(\text{MLP}_{2}(\boldsymbol{\hat{h}_{0}}))},\end{align*}

(3) \begin{align*} &[\boldsymbol{\hat{x}}_{0},\boldsymbol{\hat{h}}_{0}] = \phi_{\omega}(\mathcal{M}_{t}, t, \mathcal{P}), \end{align*}

where $\sigma (\cdot )$ is a sigmoid function, and thus the range of $\hat{v}$ lies in $[0,1]$. The large $\hat{v}$ indicates high binding affinity. The function $\phi _{\omega }$ is an SE(3)-equivariant GNN with $L$ layers, and the $(\ell +1)$th layer is computed as follows: 


(4) \begin{align*} &\boldsymbol{h_{i}^{\ell+1}} = \boldsymbol{h_{i}^{\ell}} + \sum_{j\in \mathcal{V}, i\neq j} f_{h}(d_{ij}^{\ell}, \boldsymbol{h_{i}^{\ell}}, \boldsymbol{h_{j}^{\ell}}, e_{ij}),\end{align*}

(5) \begin{align*} &\boldsymbol{x_{i}^{\ell+1}} = \boldsymbol{x_{i}^{\ell}} + \sum_{j\in \mathcal{V}, i\neq j} (\boldsymbol{x_{i}^{\ell}} - \boldsymbol{x_{j}^{\ell}})f_{x}(d_{ij}^{\ell}, \boldsymbol{x_{i}^{\ell}}, \boldsymbol{x_{j}^{\ell}}, e_{ij}) \odot \widetilde{\mathcal{M}}_{lig}, \end{align*}

where $d_{ij}^{\ell } = \|\boldsymbol{x_{i}}^{\ell } - \boldsymbol{x_{j}}^{\ell } \|$ represents the Euclidean distance between atom $i$ and atom $j$ at the $\ell $th layer, and $\boldsymbol{e_{ij}}$ indicates the direction of message-passing, including from protein to protein, from protein to ligand, from ligand to protein, and from ligand to ligand. The functions $f_{h}$ and $f_{x}$ are graph attention networks. The $\widetilde{\mathcal{M}}_{lig}$ is the mask matrix for the ligand to keep the coordinates of the protein atoms fixed. We denote $\boldsymbol{\hat{h}_{0}} = [\boldsymbol{h_{0}^{L}},...,\boldsymbol{h_{N_{M}-1}^{L}}]$ to represent all the learned atom type embeddings by Eq. (4), and it is fed into the Eq. (2) for the denoising prediction and the affinity prediction.

Since the distance $d_{ij}$ remains unchanged under translation and rotation operations, and the initial values of $\boldsymbol{h_{i}}$, $\boldsymbol{h_{j}}$, and $\boldsymbol{e_{ij}}$ are obtained from atom type and edge features, which are also invariant to SE(3)-transformations, we can conclude that the way of updating node information in Eq. (4) maintains $\boldsymbol{h_{i}^{l}}$ as SE(3)-invariant. Thus, $\hat{v} = \frac{1}{N_{M}}\sum _{i=0}^{N_{M}-1} \sigma (\text{MLP}_{2}(\boldsymbol{\hat{h}_{0}}))$ is SE(3)-invariant, which is reasonable since translating and rotating the protein–ligand complex should not change the binding affinity.

Finally, we train our model by optimizing the loss function as follows: 


(6) \begin{align*}& \mathcal{L} = \|\boldsymbol{x_{0} - \hat{x}_{0}}\|^{2} + \gamma \text{KL}(c(\boldsymbol{z_{t}}, \boldsymbol{z_{0}}) \| c(\boldsymbol{z_{t}}, \boldsymbol{\hat{z}_{0}})) + \lambda \|v - \hat{v}\|^{2},\end{align*}

where $\lambda $ and $\gamma $ are scaling factors, and $c(\cdot )$ is a categorical distribution. Notice that if we set $\lambda =0$ and remove the branch for the affinity prediction in $\phi _{\theta }$, our method degenerates back to TargetDiff [13]. The additional loss term $\|v - \hat{v}\|^{2}$ in Eq. (6) trains the affinity branch to become an expert network for the binding affinity computation, and its prediction performance is controlled by minimizing the fitting error, with the domain knowledge conveyed from the Vina affinity terms to the expert network in $\phi _{\theta }$. Moreover, the affinity fitting provides another learning force to update the parameters in the shared network $\phi _{\omega }$, and help the denoising process to extract high-quality, target-aware features, generating molecules with high binding affinity with the target protein.

In the literature, many deep learning methods have been proposed to address the problem of predicting the binding affinity between proteins and ligands, which is a crucial task in target-aware drug design [17–21]. However, these methods cannot be directly adopted here, because they require the input to be chemically valid molecules. Valid molecules may not be constructed from a noisy state during the diffusion process, rendering these methods incompatible with the diffusion framework. In contrast, our expert network in $\phi _{\theta }$ can predict the binding affinity using only the noisy information of atom types and atom coordinates. According to Eq. (1)&(2), the affinity prediction is computed not from the generated molecules, but from the atom type embeddings, which is naturally computed by Eq. (4) during the denoising process. Therefore, with the aid of the trained expert network, the knowledge of protein–ligand binding efficiently guides the molecule generation at each time step of the denoising process, and also enhances the interpretability of the model. The whole training procedure of our KGDiff is summarized as Algorithm S1 in Supplementary Materials.

### Guiding the molecule generation by binding affinity

We propose to guide the denoising process by utilizing the trained expert network throughout the whole denoising process. As illustrated in Figure 1, our method employs an iterative denoising process to generate desired molecules. The process starts with initializing a molecule with random noise (denoted as ‘Corrupted Molecule’). At the diffusion step $t$, the neural network $\phi _{\theta }$ by Eq. (1) takes as inputs the features of atom types $\boldsymbol{z_{t}}$ and atom coordinates $\boldsymbol{x_{t}}$ in protein–ligand complex, reconstructs the molecule $\widehat{\mathcal{M}}_{0} = [\boldsymbol{\hat{x}_{0}}, \boldsymbol{\hat{z}_{0}}]$ for the denoising process, and predicts the binding affinity $\hat{v}$. There is a challenge on developing an appropriate guiding scheme for the molecule generation because the molecule is determined by both continuous coordinates and discrete atom types. In the following, we devise an effective guidance on both continuous and discrete variables simultaneously.

First, we consider the guidance on the atom coordinates $\boldsymbol{x}_{t}$. In the literature [22, 23], classifier guided diffusion sampling has been demonstrated to be effective on image synthesis, and it is efficiently realized via a conditional Gaussian transition process which is obtained from the unconditional one by shifting its mean with a computable bias. Here, based on Eq. (2), the predicted affinity score $\hat{v}$ can be regarded as the probability that the molecule binds to the protein. Define a binary label $v_{b}\in \{0,1\}$ to denote the binding status, and then we construct a classifier as $p_{\theta }(v_{b}=1 \,|\, [\boldsymbol{x_{t}}, \boldsymbol{z_{t}}], t, \mathcal{P} ) \mathrel{\mathop :}= \hat{v}$. The gradient of the $\log p_{\theta }(v_{b} \,|\, [\boldsymbol{x_{t}}, \boldsymbol{z_{t}}], t, \mathcal{P} )$ with respect to $\boldsymbol{x}_{t}$ provides the direction to improve the binding affinity and thus guide the determination of atom positions. Formally, with the guidance of the binding affinity, the atom coordinates $\boldsymbol{x}_{t-1}$ can be sampled efficiently from a conditional Gaussian transition process as follows: 


(7) \begin{align*}& \boldsymbol{x_{t-1}} \sim \mathcal{N}(\widetilde{\mu}_{t}(\boldsymbol{x_{t}}, \boldsymbol{\hat{x}_{0}}) + s\widetilde{\beta}_{t}I\nabla_{\boldsymbol{x_{t}}}\log p_{\theta}(v_{b} \mid [\boldsymbol{x_{t}}, \boldsymbol{z_{t}}], t, \mathcal{P}), \widetilde{\beta}_{t}I),\end{align*}

where $s$ is an atom coordinate gradient scale, $\widetilde{\mu }_{t}(\boldsymbol{x_{t}}, \boldsymbol{\hat{x}_{0}}) $ and $\widetilde{\beta }_{t}$ can be computed according to Eq. (S9) in Supplementary Materials.

To implement the guidance on the generation of the discrete atom types $\boldsymbol{z}$, we present a heuristic guiding scheme. Notice that if we regard $\boldsymbol{z_{t}}$ as a continuous variable, then we can compute the gradient of $p_{\theta }(v_{b} \mid [\boldsymbol{x_{t}}, \boldsymbol{z_{t}}], t, \mathcal{P} )$ with respect to $\boldsymbol{z_{t}}$. We directly apply the gradient to the one-hot encoding of atom types, with positive gradient increasing the values within the one-hot vector and negative gradient driving the values towards zero. To enable the gradients to influence the positions of zeros in the one-hot encoding, we introduced a small positive value $\boldsymbol{\delta }$ to the entire one-hot vector. The guidance on $\boldsymbol{z_{t}}$ is given as follows: 


(8) \begin{align*}& \boldsymbol{z_{t}} \longleftarrow \boldsymbol{z^{\prime}_{t}}, \quad \boldsymbol{z^{\prime}_{t}} = (\boldsymbol{z_{t}} + \boldsymbol{\delta}) \cdot e^{r\nabla_{\boldsymbol{z_{t}}}p_{\theta}(v_{b} \mid [\boldsymbol{x_{t}}, \boldsymbol{z_{t}}], t, \mathcal{P})},\end{align*}

where $r$ is a atom type gradient scale. $\boldsymbol{z_{t}}$ is replaced with the above $\boldsymbol{z^{\prime}_{t}}$ for computing the $\boldsymbol{z_{t-1}}$ in Eq. (S10). It is noted that $\boldsymbol{z^{\prime}_{t}}$ is not a one-hot vector anymore but still has positive entries which are guaranteed by Eq. (8). By implementing the guidance on both atom types and atom coordinates across the whole generation process, desired molecules with high binding affinities can be generated. Algorithm S2 in Supplementary Materials gives a detailed information about the knowledge-guided denoising process of KGDiff.

## EXPERIMENTAL RESULTS

For brevity, we include the introductions about the experimental setup and evaluation metrics in Supplementary Materials.

### Predicting the binding affinity for the noisy molecules by the expert network

**Figure 2:**
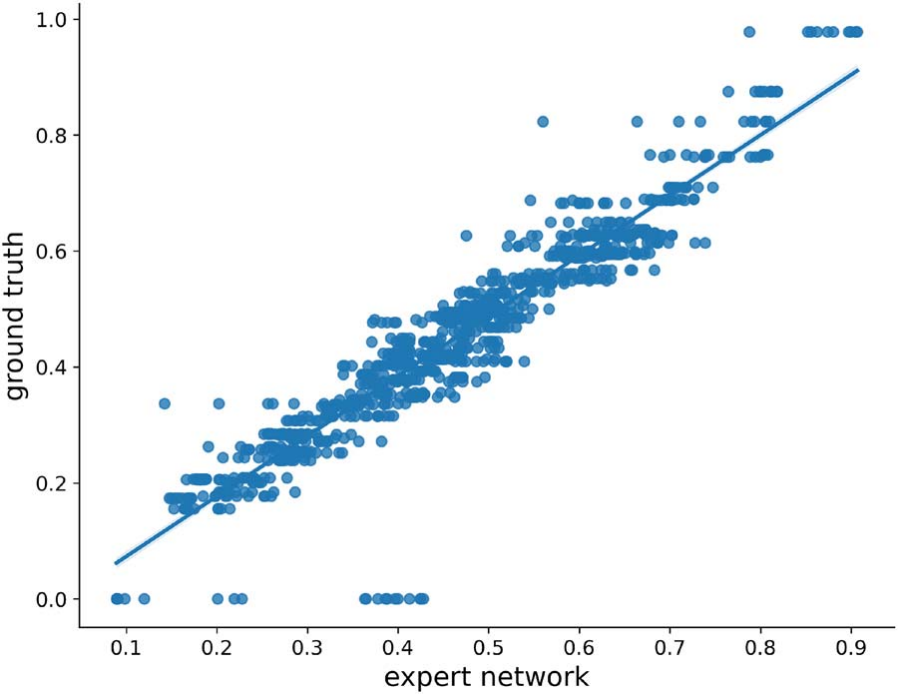
Comparisons between the expert network predictions and Vina Scores (ground truth). For each molecule, we sample $10$ steps throughout the whole denoising process with an equal interval. The Pearson correlation coefficient (Pcc) value between the ground truth and the expert network prediction is $94.0\%$.

To evaluate the performance of the expert network, we sampled 10 points throughout the denoising process at equal intervals for each protein complex in the test set. At these sampling points, we assessed the predictions of the expert network and calculated their correlation with the ground truth, i.e. the normalized Vina Scores. As illustrated in Figure 2, we observed a high correlation with a Pearson correlation coefficient (Pcc) value of up to 94.0%. To the best of our knowledge, our expert network is the first model capable of accurately predicting the binding affinity between the protein target and the noisy molecules from the denoising process.

### Binding knowledge effectively guides the molecule generation

As shown in Table 1, our method, i.e. KGDiff, outperforms the existing SOTA baselines on all binding-related metrics, including Vina Score, Vina Minimize, Vina Dock and High Affinity. Here, Vina Score is the most important metric for the task, as it directly evaluates the binding affinity of generated 3D molecules without optimizing the molecular conformation. In particular, when compared to the recent molecular diffusion model TargetDiff, KGDiff obtains a large increase by $46.2\%$ on the average of Vina Score, as well as $36.2\%$ on the median of Vina Score. This indicates the effectiveness of the knowledge guidance method in KGDiff, while TargetDiff solely depends on learning the joint distribution between the protein and the molecule. Moreover, KGDiff is the first model to outperform the reference, which is directly determined from the protein–ligand pairs in the data. Specifically, KGDiff achieves $24.4$ and $33.3\%$ improvements over the reference, in terms of the average and the median of the Vina Score, respectively. By considering from Vina Score to Vina Minimize and Vina Dock within each method, we can find that optimizing the molecular conformations locally (corresponding to Vina Minimize) and globally (corresponding to Vina Dock) can further improve the binding affinities. This phenomenon suggests that Vina Score is highly sensitive to the changes of atom coordinates.

**Table 1:** Statistical values of different molecular properties. For liGAN and GraphBP, AutoDock Vina could not parse some generated atom types when computing the Vina Score and Vina Minimize. We collect 10,000 molecules for evaluation in each method.

Method	Vina Score($\downarrow $)		Vina Minimize($\downarrow $)		Vina Dock($\downarrow $)		High Affinity($\uparrow $)		QED($\uparrow $)		SA($\uparrow $)	
	Average	Median	Average	Median	Average	Median	Average	Median	Average	Median	Average	Median
Reference	−6.46	−6.46	−6.71	−6.49	−7.45	−7.26	-	-	0.48	0.47	0.73	0.74
liGAN	-	-	-	-	−6.33	−6.20	21.1%	11.1%	0.39	0.39	0.59	0.57
GraphBP	-	-	-	-	−4.80	−4.70	14.2%	6.7%	0.43	0.45	0.49	0.48
AR	−5.75	−5.64	−6.18	−5.88	−6.75	−6.62	37.9%	31.0%	0.51	0.50	0.63	0.63
Pocket2Mol	−5.14	−4.70	−6.42	−5.82	−7.15	−6.79	48.4%	51.0%	**0.56**	**0.57**	**0.74**	**0.75**
TargetDiff	−5.50	−6.32	−6.69	−6.86	−7.83	−7.92	59.2%	60.4%	0.49	0.49	0.59	0.58
KGDiff	**−8.04**	**−8.61**	**−8.78**	**−8.85**	**−9.43**	**−9.43**	**79.2%**	**87.0%**	0.51	0.51	0.54	0.54

**Figure 3:**
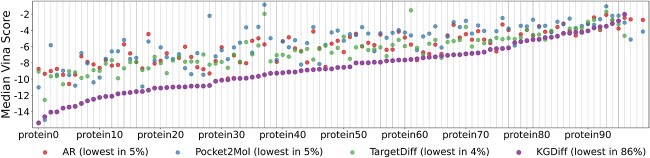
Median Vina Scores for KGDiff and other baselines across 100 proteins in the test set. The proteins are sorted by the median Vina Score of KGDiff generated molecules.

We further analyze the performance on each protein target and plot the results in Figure 3. Our model achieves the best binding affinity in $86\%$ protein targets while each of other baselines only achieves the best binding affinity in about $5\%$ protein targets. We notice that the performance of Pocket2Mol on QED and SA scores in Table 1 is currently the best. This is reasonable as our expert network does not specifically optimize against these two metrics. Moreover, in drug design, QED and SA scores are not the primary focus, and it is acceptable as long as they fall within a reasonable range. Based on the KGDiff framework, it is not difficult to explore multi-objective optimization, which could lead to further improvements on these metrics.

We carry out ablation studies demonstrating the effectiveness of training the denoising network and expert network jointly rather than training them separately. Ablation studies on the guidance for both discrete atom types and continuous atom coordinates validate the effectiveness of the designed guidance. For more detailed information, please refer to the ablation study section in the Supplementary Materials.

### Case studies demonstrate the interpretability of the expert network

To analyse the results of molecule generation, we visualize the generated molecules along with its target proteins. For fairly evaluation, we pick three target proteins where the pockets are easily identifiable and the generated molecules have high, median and low Vina Scores respectively. Specifically, to explore how our expert network evaluates individual atoms within the molecule, we visualized the scores assigned to each atom. To enhance the visibility of these scores, we applied an additional processing step by moving the center of the distribution of atomic scores to zero.

**Figure 4:**
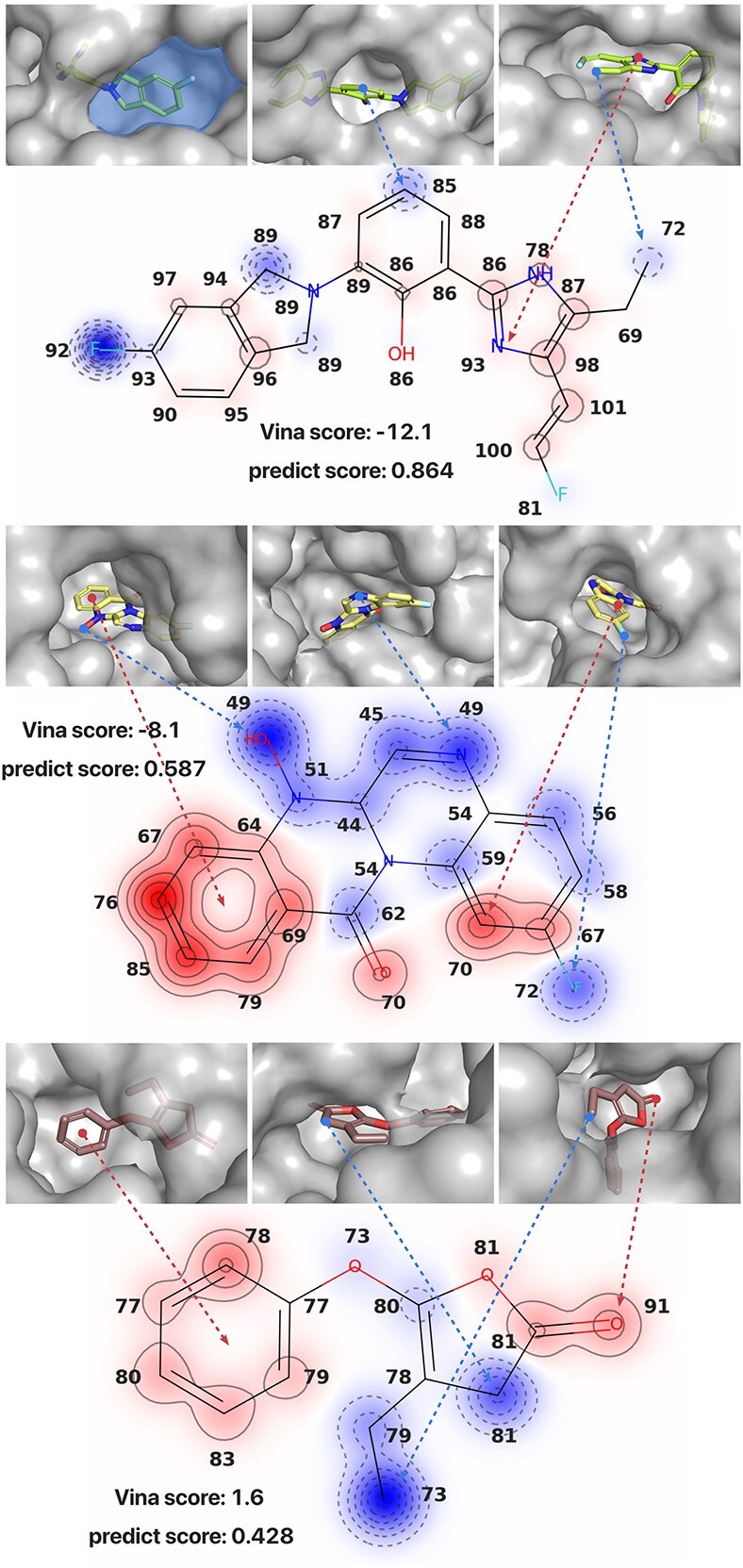
Visualization of molecular conformations in protein pockets from three camera perspectives and atomic scores within the molecule. The ‘predict score’ represents the score generated by the expert network, ranging from 0 to 1, where higher scores indicate higher binding affinities. Atoms highlighted in red indicate scores are above the average score of all atoms in the molecule, with darker red indicating higher scores. Conversely, atoms highlighted in blue have scores below the average, with darker blue indicating lower scores. The bold number around each atom indicates the number of surrounding protein atoms within 8Å.

For better clarification, let’s start by defining the ‘pocket hole interface’: it refers to the region on the surface of the protein pocket that is connected to the external environment. As illustrated in Figure 4, the light blue area in the left camera perspective of the first example is a ‘pocket hole interface’ as it separates the spatial space into two parts, i.e. the protein pocket and the external environment. It is evident that most atoms with lower scores are frequently located near the ‘pocket hole interface’ and have lower number of protein atoms surrounding themselves. For example, in the first example, we observe that the carbon atom assigned with low score at the end of the ethyl group that is connected to the imidazole ring, is situated close to the ‘pocket hole interface’ in the visualization from the right camera perspective. Conversely, the imidazole ring is located inside the pocket, leading to a relatively higher score. In the second and third examples, we also observe similar patterns, indicated by blue and red arrows, respectively.

**Figure 5:**
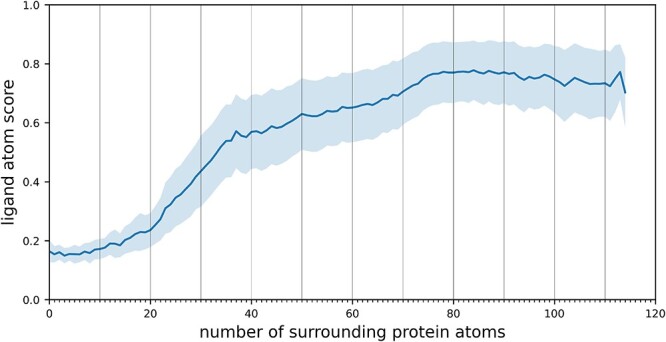
Line plot of predicted atomic scores categorized by the number of protein atoms within 8Å. The mean and variance of the scores are represented by a dark blue line and a light blue area, respectively.

We carry out an additional statistical analysis to further demonstrate the impact of protein atom density within the pocket on atomic scores. This is achieved by investigating the correlation between the scores of individual atoms and the number of protein atoms located around them within an 8-Å radius, a distance typically considered as the cutoff for non-covalent interactions in many docking programs, including Vina. As shown in Figure 5, the predicted ligand atom scores tend to increase as the number of surrounding protein atoms rises. More details can be found in Figure S2 in Supplementary Materials.

These findings can also be explained from the perspective of biochemistry. The relatively low density of protein atoms in the vicinity of the ‘pocket hole interface’ makes it less possible for ligand atoms to engage in effective non-covalent interactions, such as salt bridges and $\pi -\pi $ stacking. Conversely, atoms with higher scores are predominantly situated inside the protein pocket. These positions exhibit a higher density of protein atoms, leading to stronger protein–molecule interactions.

## THE APPLICATION SCOPE OF KGDIFF

### Generating molecules on various protein categories

We expanded molecule generation to several protein classes: GPCRs, ion channels, kinases, and phosphatases. As is shown in Table 2, KGDiff achieves the best performance on all three Vina metrics when comparing with the references. Note that even with the notably small pocket size on protein 8a1z, KGDiff still outperforms the experimental ligand. This experiment demonstrates that KGDiff can maintain high performance across a wide variety of protein categories.

**Table 2:** Statistical analysis of Vina results for four protein categories. Each pair of the protein structure and its reference ligand is acquired from experimental results. To compare with the reference ligands, KGDiff generated 100 molecules and compute the average scores across each protein target.

Category	PDBID	Vina Score($\downarrow $)		Vina Minimize($\downarrow $)		Vina Dock($\downarrow $)		Pocket
		Ref.	KGDiff	Ref.	KGDiff	Ref.	KGDiff	Volume (Å^3^)
GPCR	2rh1 [24]	−9.26	**−11.71**	−9.72	**−11.93**	−10.08	**−12.05**	171.0
Ion channel	8e56 [25]	−5.56	**−10.60**	−6.08	**−10.74**	−6.88	**−11.04**	645.9
Kinase	7jur [26]	−9.62	**−9.81**	−10.34	**−10.43**	−10.30	**−10.85**	1079.2
Phosphatase	8a1z [27]	−1.21	**−4.79**	−3.16	**−5.18**	−6.07	**−6.09**	114.3

### Pocket volumes affect the performance of KGDiff

**Figure 6:**
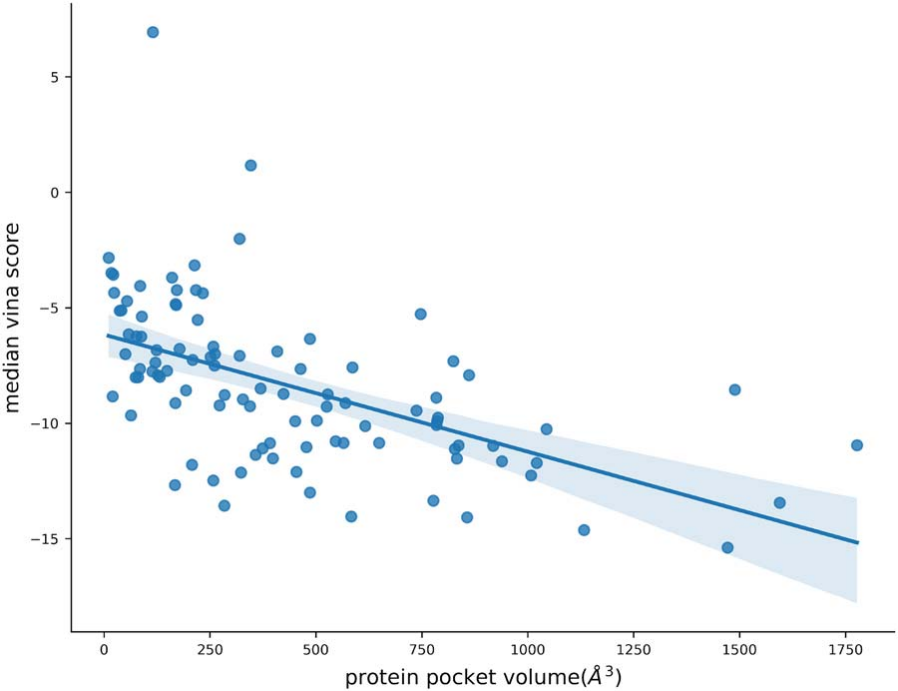
Correlation between the median Vina Scores of the generated molecules and the volumes of their corresponding protein pockets. The Pearson correlation coefficient (pcc) between Vina Scores and volumes of protein pockets is −0.545.

To assess the impact of pocket volume on the performance of KGDiff, we calculated the volume of each protein pocket in test set and examined the correlation between the Vina Scores of the molecules generated and the volumes of the corresponding protein pockets. As depicted in Figure 6, proteins with larger pockets tend to achieve better Vina Scores. Because larger pockets can accommodate more ligand atoms, increasing the likelihood of engaging in non-covalent interactions.

### Shapes of protein pockets influence the performance of KGDiff

**Figure 7:**
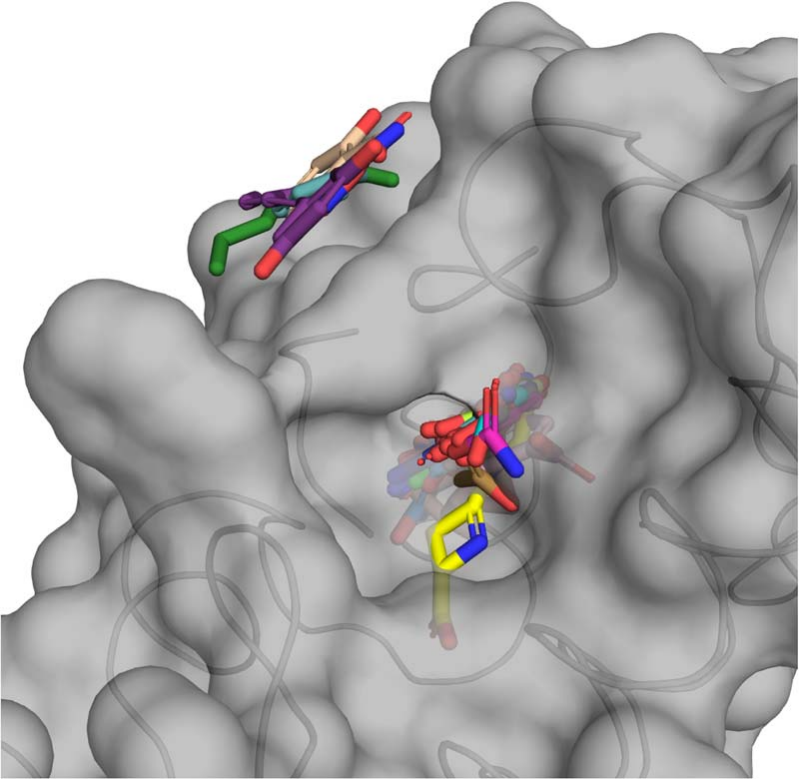
KGDiff generates molecules on both sides of the flat pocket in 2gns.

It is common for most protein pockets to possess a concave surface, which naturally accommodates ligands. As demonstrated in Table 1, under these conditions, KGDiff achieves the state-of-the-art performance. However, challenges arise when the pocket surface is relatively flat. Given that atom coordinates of all generated molecules are initialized at the center of protein pocket with the Gaussian noise, the denoising process struggles to distinguish the appropriate side towards which the noisy molecule should be directed. Consequently, molecules might be generated on both sides of the pocket surface, leading to suboptimal Vina Scores. This phenomenon is evident on the case of protein with PDBID 2gns. As is shown in Figure 7, the correct pocket is situated on the protein’s external surface, which is relatively flat. As a result, KGDiff generates molecules on both sides of the surface, yielding a median Vina Score of +38 for these molecules. We aim to address this limitation in our future work.

### Case studies on proteins of different subtypes

To assess the capability of KGDiff in generating molecules with protein subtype selectivity, an experiment was specifically conducted on the Tankyrases protein, focusing on two subtypes, TNKS1 and TNKS2. The molecules generated were used for docking to three protein groups: TNKS1, TNKS2, and all other proteins in the test set (denoted as the baseline). As demonstrated in Table 3, when we dock molecules to the subtype of the original protein, the resulting docking scores are worse than docking to the original proteins, while docking to the baseline proteins yields the worst scores. This observation is rational as protein subtypes generally share similar 3D structures with the original proteins, whereas baseline proteins exhibit more varied 3D structures.

**Table 3:** Median docking scores of re-docking and cross-docking for the test set. $\mathcal{M}_{\text{TNKS1}}$ and $\mathcal{M}_{\text{TNKS2}}$ denote the sets of molecules generated for proteins TNKS1 and TNKS2, respectively. $\mathcal{P}_{\text{TNKS1}}$ and $\mathcal{P}_{\text{TNKS2}}$ represent the target proteins TNKS1 and TNKS2, and $\mathcal{P}_{\text{baseline}}$ represent all other proteins in test set.

	$\mathcal{P}_{\text{TNKS1}}$	$\mathcal{P}_{\text{TNKS2}}$	$\mathcal{P}_{\text{baseline}}$
$\mathcal{M}_{\text{TNKS1}}$	**−15.32**	−9.37	−9.06
$\mathcal{M}_{\text{TNKS2}}$	−8.82	**−13.61**	−7.83

As shown in Table 4, we further computed the Tanimoto similarity [28] between different groups. We define the similarity between two groups as the average similarity of all distinct pairs drawn from these groups. We can observe that molecules within the same group exhibit higher similarity compared to those in the subtype group, and the lowest similarity is observed with molecules in the baseline group. Overall, the case studies on two different subtypes of Tankyrases protein demonstrate that KGDiff can generate molecules with protein subtype selectivity.

**Table 4:** Tanimoto Similarity between different sets of molecules. $\mathcal{M}_{\text{TNKS1}}$ and $\mathcal{M}_{\text{TNKS2}}$ represent generated molecules for the target proteins TNKS1 and TNKS2, and $\mathcal{M}_{\text{baseline}}$ represent generated molecules for all other proteins in test set.

	$\mathcal{M}_{\text{TNKS1}}$	$\mathcal{M}_{\text{TNKS2}}$	$\mathcal{M}_{\text{baseline}}$
$\mathcal{M}_{\text{TNKS1}}$	**15.5%**	12.2%	11.9%
$\mathcal{M}_{\text{TNKS2}}$	12.2%	**14.2%**	11.4%

## DISCUSSION

In this study, we present a novel approach for protein target-aware de novo molecule generation. By employing an expert network specifically designed to learn domain knowledge of binding affinity at every denoising step, we steer the molecule generation process towards high binding affinity. Additionally, we conduct a thorough analysis of the generated results, uncovering the underlying mechanism learned by the expert network. Experimental evaluation on the CrossDocked dataset provides compelling evidence of the effectiveness of our model, highlighting its potential in the realm of protein structure based drug design. Moreover, we demonstrate that KGDiff exhibits a preference for large pockets with concave surfaces and possesses the capability to generate molecules with protein subtype selectivity. Additionally, our approach can be extended to optimize generated molecules for more specific desired properties, such as QED and SA, by training corresponding expert networks on those properties.

However, it is important to acknowledge the limitations of our model and identify areas for future improvement. Firstly, our current approach independently adds noise to the features of atom types and atom coordinates, overlooking the inherent dependencies between these variables in 3D space. Secondly, the construction of chemical bonds between atoms is not explicitly modeled, and a post-processing algorithm is employed to build the generated 3D points into molecules. This may result in suboptimal molecular conformations and the generation of molecules with large rings. Thirdly, it is essential to incorporate additional chemical knowledge into the model, such as salt bridges and $\pi -\pi $ stacking. These specific non-covalent interactions play a crucial role in ligand binding and should be explicitly considered in the model. Lastly, KGDiff achieves limited performance in generating molecules for protein pockets with flat surfaces. Addressing these limitations will be a focus of our future work.

**Key Points**

- We introduce domain knowledge, i.e. the binding affinity, into the diffusion framework to guide protein structure-based molecule generation.
- Our works provide an atom-level explanation of the generated molecules from the perspective of a generative model and establish connections with domain knowledge.
- Experimental results on CrossDocked2020 [7] demonstrate the superiority of our approach over five representative baselines.

## Supplementary Material

bib2023_rebuttal_sup_bbad435

## Data Availability

All relevant data that support the findings of this work are available on Zenodo at https://zenodo.org/records/8419944.

## CODE AVAILABILITY

All relevant codes that support the findings of this work are available on GitHub at https://github.com/CMACH508/KGDiff.

## Author Biographies

**Hao Qian** is a Ph.D. candidate in the Department of Computer Science and Engineering at Shanghai Jiao Tong University (SJTU). His research interests include deep learning and de novo drug design.

**Wenjing Huang** is a Ph.D. candidate in the Department of Computer Science and Engineering at Shanghai Jiao Tong University (SJTU). Her research interests include machine learning and computer vision.

**Shikui Tu**, a tenured associate professor in the Department of Computer Science and Engineering at Shanghai Jiao Tong University (SJTU). His research interests include machine learning and bioinformatics. He has published more than 80 academic papers in top conferences and journals, including Science, Cell, Nature Communications, and NeurIPS, ICML, TNNLS etc.

**Lei Xu**, is a Zhiyuan Chair Professor of Computer Science and Engineering Department, chief scientist of AI Research Institute, chief scientist of Brain Sci & Tech Research Centre, Shanghai Jiao Tong University (SJTU) and a professor of Guangdong Institute of Intelligence Science and Technology. Elected to Fellow of IEEE in 2001 and Fellow of IAPR 2002 and of European Academy of Sciences (EURASC) in 2003.
