# Supplementary material for: KGDiff: towards explainable target-aware molecule generation with knowledge guidance
Source: Brief Bioinform. 2023 Dec 1;25(1):bbad435. doi: 10.1093/bib/bbad435 (PMC10783868; doi:10.1093/bib/bbad435)
Supplement: bib2023_rebuttal_sup_bbad435 [file bib2023_rebuttal_sup_bbad435.pdf]

Supplementary Information about the Method

Computation of Binding Affinity via AutoDock Vina

In target-aware molecule generation, the key point is to utilize the domain knowledge, i.e., the binding affinity to generate molecules that can bind to the protein target well. In this study, we adopt the methodology of the widely-used molecular docking program, AutoDock Vina [1], to calculate the binding affinity. This computation is based on a weighted summation of atomic interactions, encapsulating steric effects, hydrophobic interactions among hydrophobic atoms, hydrogen bonding, and penalties on rotatable bonds.

Specifically, the surface distance d_{ij} between ligand atom i and protein atom j is firstly computed as $d_{ij} = r_{ij} - R_i - R_j$, where R_i and R_j are the van der Waals radius of atom i and atom j respectively. Then the first three terms can be computed as follows:

$$\left.\begin{aligned} gauss_1(d) &= e^{-(d/0.5)^2} \\ gauss_2(d) &= e^{-((d-3)/2)^2} \\ repulsion(d) &= \begin{cases} d^2, & d < 0 \\ 0, & d \geq 0 \end{cases} \end{aligned}\right\} \text{steric effects,} \tag{S1}$$

$$hydrophobicity(d) = \begin{cases} 1, & d < 0.5\text{Å} \\ 1.5 - d, & 0 \leq d \leq 1.5\text{Å} \\ 0, & d > 1.5\text{Å} \end{cases}, \tag{S2}$$

$$hydrogen\ bonding(d) = \begin{cases} 1, & d < -0.7\text{Å} \\ d/-0.7, & -0.7\text{Å} \leq d \leq 0 \\ 0, & d > 0 \end{cases}. \tag{S3}$$

Steric effects result from repulsive forces that occur between overlapping electron clouds, and these effects have broad applications in both theoretical and practical chemistry. Hydrophobicity is a property that describes the tendency of certain atoms to be repelled from water. Hydrogen bonding represents a unique form of dipole-dipole attraction between molecules. It is typically observed when a hydrogen atom covalently bonded to a highly electronegative atom forms an attractive force with another highly electronegative atom. Additionally, these interactions are adjusted by the number of rotatable bonds between heavy atoms in the ligand to compensate for entropic penalties.

Preliminaries

Our model is a diffusion model, and it constitutes a latent variable model which can be described as $p_\theta(\mathcal{M}_0|\mathcal{P}) = \int p_\theta(\mathcal{M}_{0:T}|\mathcal{P})d\mathcal{M}_{1:T}$, where $\mathcal{M}_{1:T} = \mathcal{M}_1, \mathcal{M}_2, \ldots, \mathcal{M}_T$ represent a sequence of latent variables. These variables share the same dimensionality as the data $\mathcal{M}_0$, which is assumed to be distributed as $\mathcal{M}_0 \sim p(\mathcal{M}_0|\mathcal{P})$. The diffusion model consists of a forward process (or diffusion process), in which a molecule is progressively added noise, and a reverse process (or denoising process), in which noise is transformed back into a sample from the target distribution. Both process are defined as Markov chains and can be represented with a neural network parameterized by θ as follows:

$$q(\mathcal{M}_{1:T}|\mathcal{M}_0, \mathcal{P}) = \prod_{t=1}^{T} q(\mathcal{M}_t|\mathcal{M}_{t-1}, \mathcal{P}), \tag{S4}$$

$$p_\theta(\mathcal{M}_{0:T-1}|\mathcal{M}_T, \mathcal{P}) = \prod_{t=1}^{T} p_\theta(\mathcal{M}_{t-1}|\mathcal{M}_t, \mathcal{P}). \tag{S5}$$

In the following, we follow the previous work [2] and review the diffusion model as the background.

Molecular Diffusion Process

The distribution of molecules can be represented by the production of continuous atom coordinate distribution modeled with a Gaussian distribution $\mathcal{N}$ and discrete atom type distribution modeled with a categorical distribution $\mathcal{C}$. Then each step t in forward Markov diffusion process can be formulated with fixed variance schedules $\beta_1, \ldots, \beta_T$:

$$\begin{aligned} q(\mathcal{M}_t|\mathcal{M}_{t-1}, \mathcal{P}) = \mathcal{N}(\boldsymbol{x_t}; \sqrt{1-\beta_t}\boldsymbol{x_{t-1}}, \beta_t \boldsymbol{I}) \times \\ \mathcal{C}(\boldsymbol{z_t}|(1-\beta_t)\boldsymbol{z_{t-1}} + \beta_t/K). \end{aligned} \tag{S6}$$

While the schedules in the two distributions may differ in practice, we utilize the same symbol to denote them for brevity. The joint distribution of molecules is broken down into the product of two independent distributions: namely, the atom coordinate distributions and the atom type distributions. This approach is advantageous because these independent distributions provide straightforward mathematical formulations, and consequently, noisy samples can be efficiently generated.

We denote $\alpha_t = 1 - \beta_t$ and $\overline{\alpha}_t = \prod_{s=1}^{t} \alpha_s$, then the noisy data distribution $q(\mathcal{M}_t|\mathcal{M}_0)$ at any time step t can be computed in a closed-form expression:

$$q(\boldsymbol{x_t}|\boldsymbol{x_0}) = \mathcal{N}(\boldsymbol{x_t}; \sqrt{\overline{\alpha}_t}\boldsymbol{x_0}, (1-\overline{\alpha}_t)\boldsymbol{I}), \tag{S7}$$

$$q(\boldsymbol{z_t}|\boldsymbol{z_0}) = \mathcal{C}(\boldsymbol{z_t}|\overline{\alpha}_t \boldsymbol{z_0} + (1-\overline{\alpha}_t)/K). \tag{S8}$$

We can then compute the normal posterior of atom coordinates and categorical posterior of atom types in a closed-form solution by leveraging the Bayes theorem:

$$q(\boldsymbol{x_{t-1}}|\boldsymbol{x_t}, \boldsymbol{x_0}) = \mathcal{N}(\boldsymbol{x_{t-1}}; \widetilde{\mu}_t(\boldsymbol{x_t}, \boldsymbol{x_0}), \widetilde{\beta}_t \boldsymbol{I}), \tag{S9}$$

$$q(\boldsymbol{z_{t-1}}|\boldsymbol{z_t}, \boldsymbol{z_0}) = \mathcal{C}(\boldsymbol{z_{t-1}}|\widetilde{c}_t(\boldsymbol{z_t}, \boldsymbol{z_0})), \tag{S10}$$

where $\widetilde{\mu}_t(\boldsymbol{x_t}, \boldsymbol{x_0}) = \frac{\sqrt{\overline{\alpha}_{t-1}}\beta_t}{1-\overline{\alpha}_t}\boldsymbol{x_0} + \frac{\sqrt{\alpha_t}(1-\overline{\alpha}_{t-1})}{1-\overline{\alpha}_t}\boldsymbol{x_t}, \widetilde{\beta}_t = \frac{1-\overline{\alpha}_{t-1}}{1-\overline{\alpha}_t}\beta_t$, and $\widetilde{c}_t(\boldsymbol{z_t}, \boldsymbol{z_0}) = c^*/\sum_{k=1}^{K} c_k^*, c^*(\boldsymbol{z_t}, \boldsymbol{z_0}) = [\alpha_t \boldsymbol{z_t} + (1-\alpha_t)/K] \odot [\overline{\alpha}_{t-1}\boldsymbol{z_0} + (1-\overline{\alpha}_{t-1})/K]$.

Molecular Denoising Process

The denoising process is defined as reconstructing the molecule $\mathcal{M}_0$ from random noise $\mathcal{M}_T$. The reversed diffusion process is learned with a neural network ϕ parameterized by θ:

$$\begin{aligned} p_\theta(\mathcal{M}_{t-1}|\mathcal{M}_t, \mathcal{P}) = &\mathcal{N}(\boldsymbol{x_{t-1}}; \mu_\theta([\boldsymbol{x_t}, \boldsymbol{z_t}], t, \mathcal{P}), \widetilde{\beta}_t \boldsymbol{I}) \times \\ &\mathcal{C}(\boldsymbol{z_{t-1}}|c_\theta([\boldsymbol{x_t}, \boldsymbol{z_t}], t, \mathcal{P})). \end{aligned} \tag{S11}$$

There are different ways to parameterize $\mu_\theta([\boldsymbol{x_t}, \boldsymbol{z_t}], t, \mathcal{P})$ and $c_\theta([\boldsymbol{x_t}, \boldsymbol{z_t}], t, \mathcal{P})$. In this paper, we train a neural network ϕ_θ to predict $[\boldsymbol{x_0}, \boldsymbol{z_0}]$ and compute μ_θ and c_θ by leveraging Eq. (S9) and Eq. (S10), where the posteriors of atom coordinates and atom types are defined respectively.

Pseudocode for Training and Inference

We summarize the detailed training and generating process in Algorithm S1 and Algorithm S2 respectively.

Experimental Setup

Dataset Preparation

We adopt the data preparation and selection strategy in the previous works [3, 4]. The evaluation of the generation

Algorithm S1 Training Procedure of KGDiff

Input: Protein-ligand complex $\{\mathcal{P}, \mathcal{M}, v\}_{i=1}^{N}$, neural network ϕ_θ, atom coordinate loss scale γ, atom type loss scale λ
while ϕ_θ not converage **do**
  Sample diffusion step $t \in \{0, ..., T\}$
  Move the complex to make the center of mass (CoM) of protein atoms zero
  Compute $\boldsymbol{x_t}$: $\boldsymbol{x_t} = \sqrt{\overline{\alpha}_t}\boldsymbol{x_0} + (1-\overline{\alpha}_t)\boldsymbol{\epsilon}$, where $\boldsymbol{\epsilon} \in \mathcal{N}(0, \boldsymbol{I})$
  Compute $\boldsymbol{z_t}$:
    $\log \boldsymbol{c} = \log(\overline{\alpha}_t \boldsymbol{z_0} + (1-\overline{\alpha}_t)/K)$,
    $\boldsymbol{z_t} = one\_hot(\arg\max_i [g_i + \log z_i])$,
    where $g \sim \text{Gumble}(0, 1)$
  Compute $[\boldsymbol{\hat{x}_0}, \boldsymbol{\hat{z}_0}, \hat{v}]$: $[\boldsymbol{\hat{x}_0}, \boldsymbol{\hat{z}_0}, \hat{v}] = \phi_\theta([\boldsymbol{x_t}, \boldsymbol{z_t}], t, \mathcal{P})$
  Compute the posterior atom types $c(\boldsymbol{z_t}, \boldsymbol{z_0})$, $c(\boldsymbol{z_t}, \boldsymbol{\hat{z}_0})$ according to equation S10
  Compute weighted loss on atom types, atom coordinates and binding affinities:
    $\mathcal{L} = \|\boldsymbol{x_0} - \boldsymbol{\hat{x}_0}\|^2 + \gamma \text{KL}(c(\boldsymbol{z_t}, \boldsymbol{z_0}) \| c(\boldsymbol{z_t}, \boldsymbol{\hat{z}_0})) + \lambda \|v - \hat{v}\|^2$
  Update θ by minimizing $\mathcal{L}$
end while

Algorithm S2 Inference Procedure of KGDiff

Input: The protein pocket $\mathcal{P}$, the trained neural network ϕ_θ, atom coordinate gradient scale s, atom type gradient scale r
Output: Generated molecule $\mathcal{M}$ with a predicted affinity v
Sample the number of atoms in $\mathcal{M}$ based on a prior distribution conditioned on the pocket size
Move the CoM of protein to zero using a translation vector denoted as $\triangle \boldsymbol{x_p}$
Initialize atom coordinates x_T and atom types z_T:
  $\boldsymbol{x_T} \sim \mathcal{N}(0, \boldsymbol{I})$,
  $\boldsymbol{z_T} = one\_hot(argmax_i g_i)$, where $g \sim Gumble(0, 1)$
for $t = T$ to 1 **do**
  Predict $[\boldsymbol{\hat{x}_0}, \boldsymbol{\hat{z}_0}, \hat{v}]$: $[\boldsymbol{\hat{x}_0}, \boldsymbol{\hat{z}_0}, \hat{v}] = \phi_\theta([\boldsymbol{x_t}, \boldsymbol{z_t}], t, \mathcal{P})$
  Compute $\widetilde{\mu}_t(\boldsymbol{x_t}, \boldsymbol{\hat{x}_0})$ and $\widetilde{\beta}_t$ according to equation S9
  Sample $\boldsymbol{x_{t-1}}$ with affinity guidance: $\boldsymbol{x_{t-1}} \sim \mathcal{N}(\widetilde{\mu}_t(\boldsymbol{x_t}, \boldsymbol{\hat{x}_0}) + s\widetilde{\beta}_t I \nabla_{\boldsymbol{x_t}} \log p_\theta(v_b \mid [\boldsymbol{x_t}, \boldsymbol{z_t}], t, \mathcal{P}), \widetilde{\beta}_t I)$
  Guide $\boldsymbol{z_t}$ with binding affinity:
    $\boldsymbol{z'_t} = (\boldsymbol{z_t} + \boldsymbol{\delta}) \cdot e^{r \nabla_{\boldsymbol{z_t}} p_\theta(v_b | [\boldsymbol{x_t}, \boldsymbol{z_t}], t, \mathcal{P})}$,
    where the constant $\boldsymbol{\delta} \to \boldsymbol{0}^+$
  Sample $\boldsymbol{z_{t-1}}$ according to equation S10:
    $\boldsymbol{z_{t-1}} \sim \mathcal{C}(\boldsymbol{z_{t-1}} | \widetilde{c}_t(\boldsymbol{z'_t}, \boldsymbol{\hat{z}_0}))$
end for
return $[\boldsymbol{x_0} + \triangle \boldsymbol{x_p}, \boldsymbol{z_0}], \hat{v}$

performance was conducted using the CrossDocked dataset [5], which is a protein-ligand database specifically designed for deep learning methods. We applied certain criteria for data filtering, including excluding poses with a root mean square deviation (RMSD) of tied localization poses greater than 1 Å and proteins with more than 30% sequence identity. To facilitate the learning of binding knowledge by our model, we normalized the binding affinity values in the CrossDocked dataset to a range of $[0, 1]$ where higher values correspond to better binding affinity. Finally, we obtained approximately 100,000 complexes for training and 100 complexes for testing.

Baselines

We have included five generative models as baselines which can be divided into three categories:

- CNN based model: LiGAN [5]
- auto-regressive models: AR [3], Pocket2Mol [4], GraphBP [6]
- diffusion-based models: TargetDiff [2]

Metrics

To provide a comprehensive evaluation of all the generative methods considered, we assess the performance of our model and baselines based on the following metrics:

- Vina Score: The Vina Score refers to the scoring function employed in AutoDock Vina [1], a popular molecular docking software program utilized by many prevailing deep generative models as a evaluation tool. The Vina Score is a numerical value that reflects the predicted binding energy between the ligand and the receptor, with lower scores indicating stronger binding affinities.
- Vina Minimize: The “Minimize” operation is used for energy minimization of a ligand molecule. It optimizes the ligand’s geometry by reducing its potential energy through an iterative process. The goal of Vina minimize is to refine the ligand’s conformation and prepare it for subsequent docking simulations. It does not involve the docking process itself but focuses solely on the ligand’s structure.
- Vina Dock: The “Dock” command is used for molecular docking, which predicts the optimal binding conformation and affinity between a ligand molecule and a target protein. Vina dock performs a systematic search of the ligand’s conformational space within the protein’s binding site and evaluates the binding affinity using a scoring function. It generates multiple docking poses and ranks them based on their predicted binding affinity. The purpose of Vina dock is to identify potential ligand-protein interactions and predict the most favorable binding mode.
- High Affinity: High Affinity refers to the proportion of generated molecules that exhibit a higher Vina docking score to the target pockets compared to the reference molecules.
- QED (Quantitative Estimate of Drug-likeness) [7]. The QED is used to evaluate the drug-likeness of a small molecule compound, taking into account factors such as molecular weight, lipophilicity, and polar surface area. The QED score ranges from 0 to 1, with higher scores indicating better drug-likeness.
- SA (Synthetic Accessibility) [8]. The SA score is a metric used to indicate the level of difficulty in synthesizing a molecule, with values ranging from 0 to 1. A higher SA score indicates that the compound is easier to synthesize.

Additional Evaluations

Our work focuses on generating molecules with high binding affinities, and other evaluations of molecular properties are included in this section.

We evaluate the bond length distributions and ring size distributions as follows:

- Ring size distribution. The size of rings plays an important role in drug design. In the FDA Orange Book before January 2020, the most common ring systems are five-membered rings and six-membered rings[9].

Table S1. Ring size distributions of the generated molecules from each baseline and our model.

Ring Size	Reference	liGAN	AR	Pocket2Mol	TargetDiff	KGDiff
3	1.7%	28.1%	29.6%	0.1%	0.0%	0.0%
4	0.0%	15.7%	0.0%	0.0%	2.8%	2.9%
5	30.2%	29.8%	16.1%	16.4%	30.5%	21.3%
6	67.4%	22.7%	51.4%	80.4%	51.3%	46.0%
7	0.7%	2.6%	1.7%	2.6%	12.0%	21.2%
8	0.0%	0.8%	0.7%	0.3%	2.6%	6.4%
9	0.0%	0.3%	0.5%	0.1%	0.8%	2.2%

Table S2. P-values of T-test between KGDiff and KG-TargetDiff. Here, KG-TargetDiff denotes that we utilize an independently trained expert network to guide TargetDiff to generate molecules. We generate 10,000 molecules for evaluation in each method.

Method	Vina Score(↓)		Vina Minimize(↓)		Vina Dock(↓)	
	Average	Median	Average	Median	Average	Median
KG-TargetDiff	-7.50	-8.16	-8.40	-8.52	-9.20	-9.16
KGDiff	**-8.04**	**-8.61**	**-8.78**	**-8.85**	**-9.43**	**-9.43**
P-values	$2.9 \times 10^{-5} (\ll 0.05)$		$6.0 \times 10^{-6} (\ll 0.05)$		$4.5 \times 10^{-3} (\ll 0.05)$	

- E_{JSD}: The Jensen-Shannon divergence (JSD) [10] is used to measure the difference between the bond length distributions of molecules generated by different models and those in the test set. E_{JSD} is calculated as follows:

$$E_{JSD} = \sum_{b \in \mathcal{B}} \frac{N_b}{N_{\mathcal{B}}} JSD(b)$$

Here, $\mathcal{B}$ represents the set of all bond types, $N_{\mathcal{B}}$ is the total number of bonds in generated set, and $JSD(b)$ is the distance between the distribution of bond type b in the test set and the generated set. A smaller value of E_{JSD} indicates that the generated molecules have bond lengths that are more similar to those found in real compounds.

As shown in Table S1, the majority of rings (67.3%) in the molecules generated by our method are five-membered and six-membered, while a smaller portion (21.2%) consists of seven-membered rings. We attribute the higher prevalence of larger rings to limitations imposed by the post-processing algorithm. To mitigate this issue, future research will explore direct prediction of chemical bonds as an alternative approach, aiming to enhance the generation of diverse ring sizes.

Table S7 presents the Jensen-Shannon divergence (JSD) of different bond types in each baseline method and our proposed model. The bond length distribution of the molecules generated by our method exhibits a close resemblance to that of the state-of-the-art method (TargetDiff). This indicates that our guidance approach effectively maintains the bond length distribution of the molecules, thereby validating the efficacy of our guidance method.

Abaltion Studies

To investigate the impact of joint training of the denoising network and the expert network, we conducted an additional experiment by training an independent expert network with the same architecture as the expert network in KGDiff. We then used this independent expert network to guide the TargetDiff model in molecule generation. As illustrated in Table S2, our method achieves SOTA performance on all metrics with statistical significance. This outcome highlights the effectiveness of joint training compared to training the denoising network and expert network separately.

Table S3. Analysis on the effect of atom type guidance and atom coordinate guidance. For evaluation, we generated 100 molecules on each protein in the test set, leading to a total of 10,000 molecules for assessment in each method.

Method	Vina Score(↓)	
	Average	Median
TargetDiff	-5.50	-6.32
KGDiff w/o guidances	-4.76	-6.21
with coordinate guidance	-7.76 (63.0%↓)	-8.48 (36.6%↓)
with both guidances	**-8.04 (68.9%↓)**	**-8.61 (38.6%↓)**

To gain a deeper understanding of how the guidance affects the molecule generation process, we conducted a specially designed experiment. We utilized our trained expert network to predict the scores of intermediate molecules generated by TargetDiff, KGDiff, and its inferior versions on all 100 proteins in the test set throughout the entire denoising process. The corresponding trajectories are visualized in Figure S1. We observed that all curves start very close to the vertical axis, which is reasonable since all molecules are initialized from random noises. However, under different guidances or no guidance, the corresponding score curves of intermediate molecules behave differently. Notably, the scores of intermediate molecules generated by KGDiff are the highest, thanks to the guidance on both atom types and atom coordinates. When comparing the curves of various versions of KGDiff, we found that our model is more sensitive to changes in atom coordinates than in atom types. As the expert network is trained on Vina Score, this observation aligns with our previous finding that Vina Score is highly sensitive to changes in atom coordinates.

To figure out how the guidance affects the final Vina Score, we conduct a comprehensive analysis of the impact of atom type guidance and atom coordinate guidance. The results, as shown in Table S3, clearly demonstrate the positive effect of incorporating both types of guidance. Additionally,

Table S4. P-values of T-test between KGDiff($\hat{h}_0, \mathcal{P}$) and KGDiff($\hat{h}_0$) on Vina Score, Vina Minimize and Vina Dock. KGDiff($\hat{h}_0, \mathcal{P}$) denotes that we train the expert network based on $\hat{h}_0$ and $\mathcal{P}$ which are the learned features of all atoms in the protein-ligand complex while the expert network in KGDiff($\hat{h}_0$) is trained only based on $\hat{h}_0$ which are the learned features of atoms in the ligand. We generated 100 molecules for each protein in the test set, yielding a total of 10,000 molecules for evaluation in each method.

Method	Vina Score(↓)		Vina Minimize(↓)		Vina Dock(↓)	
	Average	Median	Average	Median	Average	Median
KGDiff($\hat{h}_0, \mathcal{P}$)	-7.93	-8.81	-8.87	-9.03	-9.46	-9.52
KGDiff($\hat{h}_0$)	-8.04	-8.61	-8.78	-8.85	-9.43	-9.43
P-values	0.32(>0.05)		0.17(>0.05)		0.61(>0.05)	

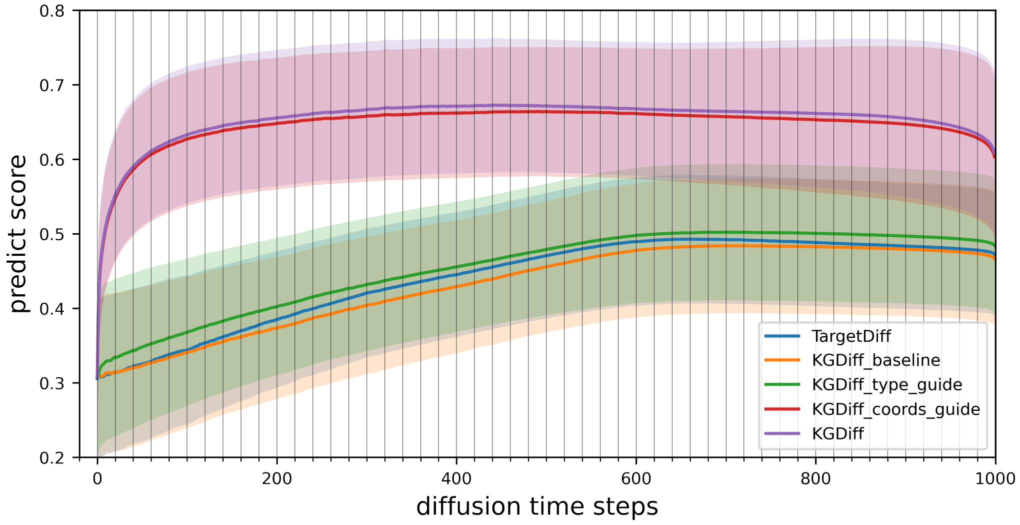

Fig. S1. Trajectories of predict scores throughout the whole diffusion process in TargetDiff and different versions of KGDiff. The mean and variance of the scores are represented by a darker line and a lighter area, respectively.

we again find that atom coordinate guidance plays a crucial role in enhancing the Vina Score, suggesting that the model is highly sensitive to changes in atom coordinates. Both curves in Figure S1 and statistic data in Table S3 provide compelling evidence of the contribution of our guidance mechanisms to the overall performance improvement. Notice that KGDiff is trained with the additional constraint of binding knowledge through the expert network, while the generation process without guidance (i.e., without incorporating the binding knowledge) does not benefit from this valuable knowledge. As a result, it is reasonable to observe that KGDiff without guidances exhibits inferior performance compared to TargetDiff.

Moreover, we ascertain that training the expert network solely on the learned ligand atom features (referred to as KGDiff($\hat{h}_0$)) is sufficient, eliminating the need to train on features of all the protein-ligand complex atoms (referred to as KGDiff($\hat{h}_0, \mathcal{P}$)). As demonstrated in Table S4, we perform a T-test on Vina Score, Vina Minimize, and Vina Dock, comparing KGDiff($\hat{h}_0$) and KGDiff($\hat{h}_0, \mathcal{P}$). P-values across these three metrics suggest that there are no significant performance differences between the two models.

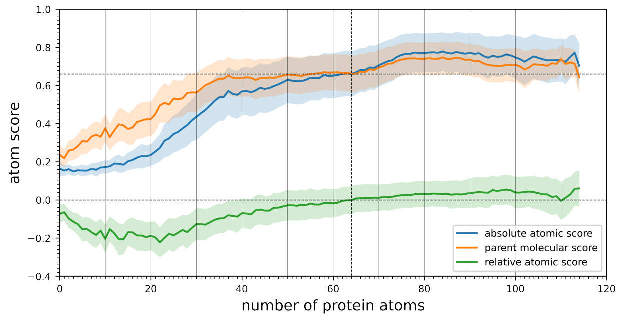

Fig. S2. Line plot of three kinds of scores. We classify all atoms from the generated molecules based on the number of protein atoms surrounding them within 8 Å. Each atom is associated with three distinct scores: the absolute atomic score, the parent molecular score, and the relative atomic score. KGDiff directly predicts the absolute atomic score, while the parent molecular score represents the average of predicted scores for all atoms within a molecule. The relative atomic score is then calculated by taking the difference between the first two scores.

Moving Towards Real-world Protein-ligand Complexes.

In this section, we tried to train KGDiff on PDBBind2020[11] which offers a comprehensive collection of protein-ligand complexes found in the Protein Data Bank (PDB) along

Training set	Protein-ligand Pairs	Unique Proteins	Unique Ligands
CrossDocked2020	99,990	14,628	8,761
PDBBind2020	14,000	14,000	11,340

Table S5. Number of proteins and ligands in two protein-ligand datasets. Ligands are considered identical if they share the same Canonical SMILES representation. Proteins are regarded identical if they share the same PDBID.

with experimentally measured binding affinity data. Training on experimentally measured binding affinities poses greater challenges compared to training on binding affinities derived from theoretical calculations. While the experimental results do not meet our expectations, we provide them here for interested readers. As illustrated in Figure S3 and Figure S4, there is a notable difference in the Pearson correlation coefficient (Pcc) when KGDiff is trained on two different datasets. Specifically, KGDiff achieves a performance of 94.0% on CrossDocked2020. However, this drops to 62.2% when trained on PDBBind2020. Here, we provide two possible explanations for this decline:

- Experimentally measured binding affinities can be influenced by various factors. First, distinct experimental methods, like Isothermal Titration Calorimetry (ITC) and Surface Plasmon Resonance (SPR), can produce somewhat divergent results. Second, environmental conditions such as temperature and pressure can also alter the outcomes. Additionally, factors like sample purity and instrument sensitivity can affect the observed affinities. In contrast, computer docking programs are unaffected by these factors. They operate based on predefined formulas for computing binding affinities, resulting in a more consistent and predictable affinity distribution.
- The data distribution of CrossDocked2020 is smoother than that of PDBBind2020. As illustrated in Table S5, both CrossDocked2020 and PDBBind2020 have comparable number of unique proteins and ligands. However, CrossDocked2020 boasts approximately 7 times more protein-ligand pairs than PDBBind2020. This difference is attributed to the cross-docking strategy, which results in a smoother data distribution for CrossDocked2020 compared to PDBBind2020. Consequently, deep learning models tend to perform better on datasets with smoother distributions.

In fact, predicting the protein-ligand binding affinity is a classic task, and many methods[12, 13, 14, 15, 16] have been proposed to address this issue. However, these methods cannot be directly adopted here, because they require the input to be chemically valid molecules. Valid molecules may not be constructed from a noisy state during the diffusion process, rendering these methods incompatible with the diffusion framework. After careful consideration, we plan to address this task in our future work.

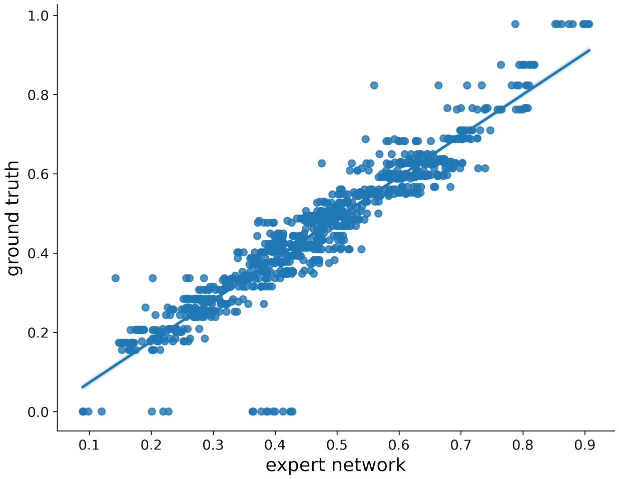

Fig. S3. Comparisons between the expert network predictions and Vina Scores (ground truth). For each molecule, we sample 10 steps throughout the whole denoising process with an equal interval. The Pearson correlation coefficient (Pcc) value between the ground truth and the expert network prediction is 94.0%.

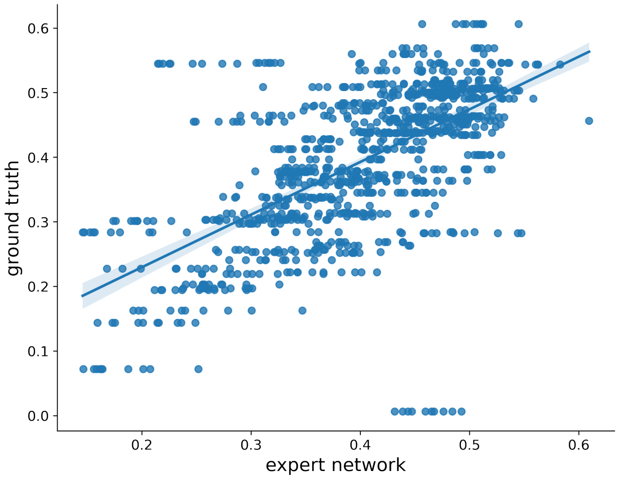

Fig. S4. Comparisons between the expert network predictions and experimentally measured binding affinities (ground truth). For each molecule, we sample 10 steps throughout the whole denoising process with an equal interval. The Pearson correlation coefficient (Pcc) value between the ground truth and the expert network prediction is 62.2%.

Related Works

Diffusion Models

Diffusion models are a prevailing class of generative models which are inspired by non-equilibrium thermodynamics [17]. A diffusion process is defined as gradually adding random noise to data through a series of diffusion steps. The key idea behind diffusion models is to learn the inverse process of the diffusion, enabling the reconstruction of desired data samples from the noise. One distinguishing feature of diffusion models is that they follow a fixed learning procedure, in contrast to other generative models such as variational autoencoders (VAEs) or flow-based models. Additionally, diffusion models have a high-dimensional latent space that is equivalent in dimensionality to the original data, offering greater expressive power.

Diffusion models have demonstrated remarkable success in generating diverse types of data, including images [18, 19] and

discrete data such as text [20, 21]. More recently, diffusion models have also been applied in the field of molecular generation and docking. For instance, GeoDiff [22] focuses on generating molecular conformations based on 2D molecular graphs. EDM [23] applies diffusion models to unconditional 3D molecule generation. Diffdock [24] utilizes a diffusion model over the non-Euclidean manifold of ligand poses and maps this manifold to the product space of translational, rotational, and torsional degrees of freedom involved in docking.

Molecular Representation

The existing molecular representations can be categorized into three types: 1D string-based, 2D graph-based and 3D structure-based. SMILES [25] is one of the most widely used 1D representations which is compatible with various natural language models such as RNNs and Transformers, and has been applied to molecular generation tasks [26, 27, 28, 29, 30, 31, 32, 33]. However, 1D representations face challenges in capturing the structural characteristics of molecules and may generate invalid molecules [34]. As a result, subsequent research has focused on using 2D molecular graph structures to learn molecular representations and generate molecules [35, 36, 37, 6, 38, 39]. Nevertheless, these methods encounter difficulties in learning the spatial information of molecules, which is crucial in target-aware molecule generation. Recently, there have been endeavors to directly generate molecules atom by atom in 3D space [40], and alternative approaches involve utilizing equivariant graph networks [41], flow-based models [42], and diffusion models [23, 43, 44, 45] to extract molecular features from 3D space and generate 3D molecules.

Protein Target-Aware Molecule Generation

In recent years, a variety of methods have been proposed for protein target-aware molecule generation. For instance, LiGANN [46] introduced a generative adversarial network to generate diverse 3D ligand shapes that complement the protein pocket and decodes them into SMILES. LiGAN [5] voxelized the 3D space of protein-ligand complexes and utilized a 3D CNN to extract atom features within a conditional VAE framework. AlphaDrug [28] employed a Lmser Transformer to learn the generation policy based on SMILES and protein sequences, enhanced by Monte Carlo Tree search. SiamFlow [47] designed a flow-based model to generate molecule graphs conditioned on protein sequences.

More recently, a wave of autoregressive methods, such as AR [3], GraphBP [6], and Pocket2Mol [4], generates molecules atom by atom in 3D protein pockets. FLAG [48] generates molecules fragment by fragment. Additionally, there are diffusion-based methods, including TargetDiff [2], DiffSBDD [49], and DiffBP [50]. These models explicitly consider SE(3)-equivariance constraints during the molecule generation process, resulting in improved performance. However, none of the methods mentioned above take into account the binding affinity between ligands and targets, which is a critical aspect of drug design. Moreover, these methods often lack interpretability when it comes to the generated results. In contrast, our models explicitly learn the binding knowledge by training an expert network, which not only improves interpretability but also enhances the overall performance.

A List of PDBIDs of the Proteins in Test Set

Here, we provide a list of PDBIDs of the proteins in test set. Corresponding protein files can be found in our github homepage: `https://github.com/CMACH508/KGDiff`.

PDBIDs							
2z3h	4aaw	4yhj	14gs	2v3r	4rn0	1fmc	3daf
1a2g	5w2g	3dzh	3g51	1coy	2jjg	2rhy	2pqw
4g3d	5bur	3gs6	1r1h	1dxo	1gg5	5q0k	5b08
2azy	5i0b	1phk	4keu	4q8b	1djy	5l1v	4zfa
2rma	3b6h	2zen	4p6p	3u5y	4f1m	4tqr	4lfu
3jyh	4iwq	1l3l	5ngz	1e8h	2e24	2hcj	3kc1
1d7j	4ja8	4u5s	4iiy	3v4t	3tym	4d7o	3ej8
1rs9	4kcq	3pdh	1umd	4pxz	2gns	1ai4	5mma
2cy0	3w83	2e6d	4rv4	5d7n	5mgl	1h36	4gvd
4tos	5aeh	4h3c	4rlu	4xli	3l3n	5tjn	5liu
3o96	4qlk	3hy9	4bel	3nfb	4m7t	3u9f	4aua
2f2c	3chc	1k9t	1h0i	4z2g	3af2	1jn2	3li4
3pnm	1afs	4azf	2pc8				

Table S6. PDBIDs of the 100 proteins in test set.

Model Parameters and Hyperparameters

Scaling Gradients

We present the evaluation results in Table S8, which includes different combinations of the gradient scale s for atom coordinates and the gradient scale r for atom types. We choose $s = 100$ and $r = 25$ based on the Vina socre. For efficiency we generate 200 molecules totally based on the protein targets in test set.

Featurization

We adopt the atom featurization approach proposed by TargetDiff [2] and provide a brief review of it for convenience. At the l-th layer, the protein-ligand complex is dynamically constructed as a k-nearest neighbors (knn) graph based on the known protein atom coordinates and known ligand atom coordinates, which are the output of the (l-1)-th layer. We choose k to be 32. The protein atom feature includes information about chemical elements, amino acid types, and whether the atoms are part of the backbone. The ligand atom types are represented as one-hot vectors, which include information about chemical element types and aromaticity. The edge features are obtained by taking the outer product of the distance embedding and the bond types. The distance embedding is generated using a set of radial basis functions centered at 20 points ranging from 0Å to 10Å. The bond type is represented as a 4-dimensional one-hot vector, indicating whether the connection is between protein atoms, ligand atoms, protein-ligand atoms, or ligand-protein atoms.

Model Parameters

The shared feature extraction module ϕ_ω comprises 9 equivariant layers, with each layer being a Transformer model having a $hidden\_dim$ of 128 and n_{head} of 16. We follow previous work[2] to choose a sigmoid β schedule with $\beta_1 = 1e-7$ and $\beta_T = 2e-3$ for atom coordinates, and a cosine β schedule with $s = 0.01$. The number of diffusion steps T is 1000.

Table S7. Jensen-Shannon divergence (JSD) of each bond type in the generated molecules from each baseline and our model. The symbols −”, =”, and “@” represent single, double, and aromatic bonds, respectively. A lower JSD value indicates better performance.

Bond	liGAN	AR	Pocket2Mol	TargetDiff	KGDiff
C−C	0.601	0.609	0.496	**0.374**	0.379
C=C	0.665	0.620	0.561	**0.500**	0.559
C@C	0.497	0.451	0.416	0.265	**0.205**
C−N	0.634	0.474	0.416	**0.365**	0.380
C=N	0.749	0.635	0.629	**0.553**	0.583
C@N	0.638	0.552	0.487	**0.224**	0.327
C−O	0.656	0.492	0.454	**0.418**	0.443
C=O	0.661	0.558	0.516	0.468	**0.435**
C@O	0.833	0.811	0.783	0.802	**0.762**
E_{JSD}	0.617	0.544	0.461	**0.348**	0.387

Training and Sampling Details

In training phase, the parameter γ is set to 100, and the parameter λ is set to 1 in Algorithm S1. The learning rate is scheduled to decay exponentially with a decay factor of 0.95, and a minimum learning rate of 1e-5 is enforced. The learning rate is decayed if there is no improvement in the validation loss for 15 consecutive evaluations.

To optimize the Vina Score during inference, we set the parameter r to 100 and the parameter s to 25 in Algorithm S2. These parameter values are chosen to obtain the best possible Vina Score according to Table S8.

We trained our model using a single NVIDIA A100 GPU (40GB) and conducted testing on twenty-four NVIDIA A100 GPUs (40GB) in parallel. We evaluate the generated molecules parallelly on 100 CPU nodes. All experiments in this paper were carried out on the Siyuan-1 cluster supported by the Center for High Performance Computing at Shanghai Jiao Tong University.

Table S8. Evaluation results for different combinations of atom coordinate gradient scale s and atom type gradient scale r.

r	s	Vina Score(↓)	valid rate (↑)	QED(↑)	SA(↑)
0.0	0.0	-5.21	71.0%	0.53	0.64
0.0	1.0	-5.61	97.5%	0.55	0.62
0.0	5.0	-6.63	97.0%	0.56	0.62
0.0	10.0	-8.04	95.5%	0.57	0.58
0.0	25.0	-7.38	69.0%	0.53	0.60
0.0	50.0	-8.26	83.5%	0.53	0.56
0.0	100.0	-7.43	66.0%	0.51	0.57
10.0	0.0	-5.64	97.0%	0.55	0.64
10.0	5.0	-8.10	96.5%	0.57	0.59
10.0	10.0	-8.07	96.5%	0.56	0.59
10.0	25.0	-8.06	89.5%	0.55	0.58
10.0	50.0	-8.16	81.5%	0.54	0.56
20.0	0.0	-5.74	97.5%	0.56	0.64
25.0	0.0	-5.68	98.0%	0.56	0.64
25.0	5.0	-8.11	96.5%	0.58	0.60
25.0	10.0	-8.25	94.5%	0.56	0.58
25.0	25.0	-7.69	90.5%	0.54	0.58
50.0	0.0	-5.78	98.0%	0.56	0.64
50.0	5.0	-8.11	96.5%	0.56	0.59
50.0	10.0	-8.18	94.5%	0.56	0.58
50.0	25.0	-8.17	91.0%	0.56	0.58
50.0	50.0	-8.22	81.0%	0.55	0.56
100.0	0.0	-5.61	73.5%	0.54	0.64
100.0	1.0	-6.65	94.0%	0.56	0.63
100.0	5.0	-8.15	95.0%	0.57	0.59
100.0	10.0	-8.19	95.5%	0.56	0.58
100.0	25.0	**-8.39**	90.0%	0.55	0.56
100.0	50.0	-7.47	83.0%	0.52	0.57
125.0	0.0	-5.88	96.0%	0.56	0.62
250.0	0.0	-5.92	91.0%	0.56	0.62
250.0	1.0	-7.01	92.5%	0.55	0.62
250.0	5.0	-7.97	95.0%	0.56	0.59
250.0	10.0	-8.09	92.5%	0.56	0.57
250.0	25.0	-8.01	90.5%	0.54	0.56
250.0	50.0	-8.12	80.5%	0.52	0.55
375.0	0.0	-5.74	85.5%	0.55	0.62
375.0	5.0	-7.97	92.0%	0.56	0.60
375.0	10.0	-8.20	88.5%	0.55	0.59
375.0	25.0	-7.73	90.5%	0.53	0.58
375.0	50.0	-8.05	77.5%	0.52	0.57
500.0	0.0	-5.59	79.0%	0.52	0.61
2500.0	0.0	-4.01	11.5%	0.38	0.52
2500.0	5.0	-3.63	16.0%	0.35	0.50

References

1. Trott, Oleg, Olson, Arthur, and J. Autodock vina: Improving the speed and accuracy of docking with a new scoring function, efficient optimization, and multithreading. *J. Comput. Chem.*, 31(2):NA–NA, 2009.
2. Jiaqi Guan, Wesley Wei Qian, Xingang Peng, Yufeng Su, Jian Peng, and Jianzhu Ma. 3d equivariant diffusion for target-aware molecule generation and affinity prediction. In *The Eleventh International Conference on Learning Representations*, 2023.
3. Shitong Luo, Jiaqi Guan, Jianzhu Ma, and Jian Peng. A 3d generative model for structure-based drug design. *Advances in Neural Information Processing Systems*, 34:6229–6239, 2021.

4. Xingang Peng, Shitong Luo, Jiaqi Guan, Qi Xie, Jian Peng, and Jianzhu Ma. Pocket2mol: Efficient molecular sampling based on 3d protein pockets. In *International Conference on Machine Learning*, pages 17644–17655. PMLR, 2022.
5. Paul G Francoeur, Tomohide Masuda, Jocelyn Sunseri, Andrew Jia, Richard B Iovanisci, Ian Snyder, and David R Koes. Three-dimensional convolutional neural networks and a cross-docked data set for structure-based drug design. *Journal of chemical information and modeling*, 60(9):4200–4215, 2020.
6. Meng Liu, Youzhi Luo, Kanji Uchino, Koji Maruhashi, and Shuiwang Ji. Generating 3d molecules for target protein binding. *arXiv preprint arXiv:2204.09410*, 2022.
7. G. Richard Bickerton, Gaia V. Paolini, and Jeremy Besnard. Quantifying the chemical beauty of drugs. *Nature Chemistry*, 4(2):90–98, 1 2012.
8. P. P. Ertl and A. Schuffenhauer. Estimation of synthetic accessibility score of drug-like molecules based on molecular complexity and fragment contributions. *Journal of Cheminformatics*, 1(1):8, 2009.
9. Jonathan Shearer, Jose L Castro, Alastair DG Lawson, Malcolm MacCoss, and Richard D Taylor. Rings in clinical trials and drugs: Present and future. *Journal of Medicinal Chemistry*, 65(13):8699–8712, 2022.
10. Frank Nielsen. On the jensen–shannon symmetrization of distances relying on abstract means. *Entropy*, 21(5), 2019.
11. Renxiao Wang, Xueliang Fang, Yipin Lu, Chao-Yie Yang, and Shaomeng Wang. The pdbbind database: methodologies and updates. *Journal of medicinal chemistry*, 48(12):4111–4119, 2005.
12. José Jiménez, Miha Skalic, Gerard Martinez-Rosell, and Gianni De Fabritiis. K deep: protein–ligand absolute binding affinity prediction via 3d-convolutional neural networks. *Journal of chemical information and modeling*, 58(2):287–296, 2018.
13. Hakime Öztürk, Arzucan Özgür, and Elif Ozkirimli. Deepdta: deep drug–target binding affinity prediction. *Bioinformatics*, 34(17):i821–i829, 2018.
14. Qichang Zhao, Fen Xiao, Mengyun Yang, Yaohang Li, and Jianxin Wang. Attentiondta: prediction of drug–target binding affinity using attention model. In *2019 IEEE international conference on bioinformatics and biomedicine (BIBM)*, pages 64–69. IEEE, 2019.
15. Kaili Wang, Renyi Zhou, Yaohang Li, and Min Li. Deepdtaf: a deep learning method to predict protein–ligand binding affinity. *Briefings in Bioinformatics*, 22(5):bbab072, 2021.
16. Dejun Jiang, Chang-Yu Hsieh, Zhenxing Wu, Yu Kang, Jike Wang, Ercheng Wang, Ben Liao, Chao Shen, Lei Xu, Jian Wu, et al. Interactiongraphnet: A novel and efficient deep graph representation learning framework for accurate protein–ligand interaction predictions. *Journal of medicinal chemistry*, 64(24):18209–18232, 2021.
17. Jascha Sohl-Dickstein, Eric Weiss, Niru Maheswaranathan, and Surya Ganguli. Deep unsupervised learning using nonequilibrium thermodynamics. In *International Conference on Machine Learning*, pages 2256–2265. PMLR, 2015.
18. Jonathan Ho, Ajay Jain, and Pieter Abbeel. Denoising diffusion probabilistic models. *Advances in neural information processing systems*, 33:6840–6851, 2020.
19. Alexander Quinn Nichol and Prafulla Dhariwal. Improved denoising diffusion probabilistic models. In *International Conference on Machine Learning*, pages 8162–8171. PMLR, 2021.
20. Jacob Austin, Daniel D Johnson, Jonathan Ho, Daniel Tarlow, and Rianne Van Den Berg. Structured denoising diffusion models in discrete state-spaces. *Advances in Neural Information Processing Systems*, 34:17981–17993, 2021.
21. Emiel Hoogeboom, Didrik Nielsen, Priyank Jaini, Patrick Forré, and Max Welling. Argmax flows and multinomial diffusion: Learning categorical distributions. *Advances in Neural Information Processing Systems*, 34:12454–12465, 2021.
22. Minkai Xu, Lantao Yu, Yang Song, Chence Shi, Stefano Ermon, and Jian Tang. Geodiff: A geometric diffusion model for molecular conformation generation. *arXiv preprint arXiv:2203.02923*, 2022.
23. Emiel Hoogeboom, Vıctor Garcia Satorras, Clément Vignac, and Max Welling. Equivariant diffusion for molecule generation in 3d. In *International Conference on Machine Learning*, pages 8867–8887. PMLR, 2022.
24. Gabriele Corso, Hannes Stärk, Bowen Jing, Regina Barzilay, and Tommi Jaakkola. Diffdock: Diffusion steps, twists, and turns for molecular docking. *arXiv preprint arXiv:2210.01776*, 2022.
25. David Weininger. Smiles, a chemical language and information system. 1. introduction to methodology and encoding rules. *Journal of chemical information and computer sciences*, 28(1):31–36, 1988.
26. Xiufeng Yang, Jinzhe Zhang, Kazuki Yoshizoe, Kei Terayama, and Koji Tsuda. Chemts: an efficient python library for de novo molecular generation. *Science and technology of advanced materials*, 18(1):972–976, 2017.
27. Daria Grechishnikova. Transformer neural network for protein-specific de novo drug generation as a machine translation problem. *Scientific reports*, 11(1):1–13, 2021.
28. Hao Qian, Cheng Lin, Dengwei Zhao, Shikui Tu, and Lei Xu. Alphadrug: protein target specific de novo molecular generation. *PNAS Nexus*, 1(4):pgac227, 2022.
29. Anvita Gupta, Alex T Müller, Berend JH Huisman, Jens A Fuchs, Petra Schneider, and Gisbert Schneider. Generative recurrent networks for de novo drug design. *Molecular informatics*, 37(1-2):1700111, 2018.
30. Francesca Grisoni, Michael Moret, Robin Lingwood, and Gisbert Schneider. Bidirectional molecule generation with recurrent neural networks. *Journal of chemical information and modeling*, 60(3):1175–1183, 2020.
31. Daniel Flam-Shepherd, Kevin Zhu, and Alán Aspuru-Guzik. Language models can learn complex molecular distributions. *Nature Communications*, 13(1):3293, 2022.
32. Xiaochu Tong, Xiaohong Liu, Xiaoqin Tan, Xutong Li, Jiaxin Jiang, Zhaoping Xiong, Tingyang Xu, Hualiang Jiang, Nan Qiao, and Mingyue Zheng. Generative models for de novo drug design. *Journal of Medicinal Chemistry*, 64(19):14011–14027, 2021.
33. Maranga Mokaya, Fergus Imrie, Willem P van Hoorn, Aleksandra Kalisz, Anthony R Bradley, and Charlotte M Deane. Testing the limits of smiles-based de novo molecular generation with curriculum and deep reinforcement learning. *Nature Machine Intelligence*, pages 1–9, 2023.
34. Wengong Jin, Regina Barzilay, and Tommi Jaakkola. Junction tree variational autoencoder for molecular graph generation. In *International conference on machine learning*, pages 2323–2332. PMLR, 2018.
35. Chence Shi, Minkai Xu, Zhaocheng Zhu, Weinan Zhang, Ming Zhang, and Jian Tang. Graphaf: a flow-based

autoregressive model for molecular graph generation. *arXiv preprint arXiv:2001.09382*, 2020.
36. Jiaxuan You, Bowen Liu, Zhitao Ying, Vijay Pande, and Jure Leskovec. Graph convolutional policy network for goal-directed molecular graph generation. *Advances in neural information processing systems*, 31, 2018.
37. Wengong Jin, Regina Barzilay, and Tommi Jaakkola. Hierarchical generation of molecular graphs using structural motifs. In *International conference on machine learning*, pages 4839–4848. PMLR, 2020.
38. Youzhi Luo, Keqiang Yan, and Shuiwang Ji. Graphdf: A discrete flow model for molecular graph generation. In *International Conference on Machine Learning*, pages 7192–7203. PMLR, 2021.
39. Meng Liu, Keqiang Yan, Bora Oztekin, and Shuiwang Ji. Graphebm: Molecular graph generation with energy-based models. *arXiv preprint arXiv:2102.00546*, 2021.
40. Niklas Gebauer, Michael Gastegger, and Kristof Schütt. Symmetry-adapted generation of 3d point sets for the targeted discovery of molecules. *Advances in neural information processing systems*, 32, 2019.
41. Vıctor Garcia Satorras, Emiel Hoogeboom, and Max Welling. E (n) equivariant graph neural networks. In *International conference on machine learning*, pages 9323–9332. PMLR, 2021.
42. Victor Garcia Satorras, Emiel Hoogeboom, Fabian B Fuchs, Ingmar Posner, and Max Welling. E (n) equivariant normalizing flows. *arXiv preprint arXiv:2105.09016*, 2021.
43. Clement Vignac, Nagham Osman, Laura Toni, and Pascal Frossard. Midi: Mixed graph and 3d denoising diffusion for molecule generation. *arXiv preprint arXiv:2302.09048*, 2023.
44. Chenqing Hua, Sitao Luan, Minkai Xu, Rex Ying, Jie Fu, Stefano Ermon, and Doina Precup. Mudiff: Unified diffusion for complete molecule generation. *arXiv preprint arXiv:2304.14621*, 2023.
45. Xingang Peng, Jiaqi Guan, Qiang Liu, and Jianzhu Ma. Moldiff: Addressing the atom-bond inconsistency problem in 3d molecule diffusion generation. *arXiv preprint arXiv:2305.07508*, 2023.
46. Miha Skalic, Davide Sabbadin, Boris Sattarov, Simone Sciabola, and Gianni De Fabritiis. From target to drug: generative modeling for the multimodal structure-based ligand design. *Molecular pharmaceutics*, 16(10):4282–4291, 2019.
47. Cheng Tan, Zhangyang Gao, and Stan Z Li. Target-aware molecular graph generation. *arXiv preprint arXiv:2202.04829*, 2022.
48. Zaixi Zhang, Yaosen Min, Shuxin Zheng, and Qi Liu. Molecule generation for target protein binding with structural motifs. In *The Eleventh International Conference on Learning Representations*, 2023.
49. Arne Schneuing, Yuanqi Du, Charles Harris, Arian Jamasb, Ilia Igashov, Weitao Du, Tom Blundell, Pietro Lió, Carla Gomes, Max Welling, et al. Structure-based drug design with equivariant diffusion models. *arXiv preprint arXiv:2210.13695*, 2022.
50. Haitao Lin, Yufei Huang, Meng Liu, Xuanjing Li, Shuiwang Ji, and Stan Z Li. Diffbp: Generative diffusion of 3d molecules for target protein binding. *arXiv preprint arXiv:2211.11214*, 2022.
